# Lokaltherapeutisches Vorgehen bei Blasen der Haut: Ein Positionspapier der Initiative Chronische Wunden (ICW) e. V.

**DOI:** 10.1007/s00105-022-05013-5

**Published:** 2022-06-09

**Authors:** Joachim Dissemond, Anke Bültemann, Veronika Gerber, Martin Motzkus, Christian Münter, Cornelia Erfurt-Berge

**Affiliations:** Klinik und Poliklinik für Dermatologie, Venerologie und Allergologie, Universitätsklinikum Essen, Hufelandstraße 55, 45122 Essen, Deutschland

**Keywords:** Erosionen, Dermatologie, Verbrennungen, Infektionen, Schmerzen, Erosions, Dermatology, Burns, Infections, Pain

## Abstract

Blasen an der Haut können durch sehr unterschiedliche Krankheitsbilder verursacht werden. Daher handelt es sich hierbei um eine interdisziplinär und interprofessionell relevante Herausforderung. Im klinischen Alltag werden derzeit verschiedene lokaltherapeutische Vorgehensweisen praktiziert. Entweder wird die Blase belassen oder die Blase wird punktiert und das Blasendach wird belassen, alternativ wird das komplette Blasendach abtragen. Jede dieser Vorgehensweisen hat potenzielle Vor- und Nachteile. Es erfolgte die Durchsicht der aktuellen Literatur und Konsensfindung durch die Expert*innen der Initiative Chronische Wunden (ICW) e. V. Folgende Vorgehensweisen werden empfohlen: unkomplizierte Blasen ohne Druckschmerz: Blasen belassen; druckschmerzhafte sowie palmar und plantar lokalisierte Blasen: Blase punktieren und Dach belassen; rupturierte Blasen ohne klinische Infektionszeichen: Reste des Blasendachs belassen; rupturierte Blasen mit klinischen Infektionszeichen: Reste des Blasendachs abtragen; Blasen bei Verbrennungen ab Grad 2a oder bei unklarer Verbrennungstiefe oder bei chemischer Verbrennung: Blasendach abtragen. Anschließend erfolgt jeweils die Anlage eines sterilen Wundverbandes. Beim Auftreten von Blasen an der Haut gibt es nicht die eine richtige lokaltherapeutische Vorgehensweise. Bei der Planung eines Behandlungskonzepts sollte die Genese der Blasen geklärt werden, ggf. sollte eine kausale Behandlung erfolgen. Die Lokaltherapie orientiert sich dann an verschiedenen individuellen Faktoren. Somit kann das gemeinsam mit den Patient*innen gewählte Vorgehen interindividuell sehr unterschiedlich sein.

## Abstract (en)

Blisters of the skin can be caused by very different diseases. Therefore, it is an interdisciplinary and interprofessionally relevant challenge. In the clinical routine different local therapeutic procedures are currently practiced. Either the blister is left in place or the blister is punctured and the blister roof is left in place; alternatively, the complete blister roof is ablated. Each of these approaches has potential advantages and disadvantages. A review of the current literature and consensus by the experts of the Initiative Chronische Wunde (ICW) e.V. was performed. The following approaches are recommended: uncomplicated blisters without pressure pain: leave blisters in place; pressure painful and palmar and plantar localized blisters: puncture blister and leave roof; ruptured blisters without clinical signs of infection: leave remnants of bladder roof; ruptured bladders with clinical signs of infection: remove remnants of the blister roof; blisters in burns of grade 2a or higher or in cases of unclear burn depth or chemical burn: remove blister roof. This is followed in each case by the application of a sterile wound dressing. There is no single correct local therapeutic procedure for blisters on the skin. When planning a therapeutic concept, the genesis of the blisters should be clarified and, if necessary, causal treatment should be given. Local therapy is then based on various individual factors. Thus, the approach chosen together with the patient can vary between individuals.

## Einleitung

**Figure 1:**
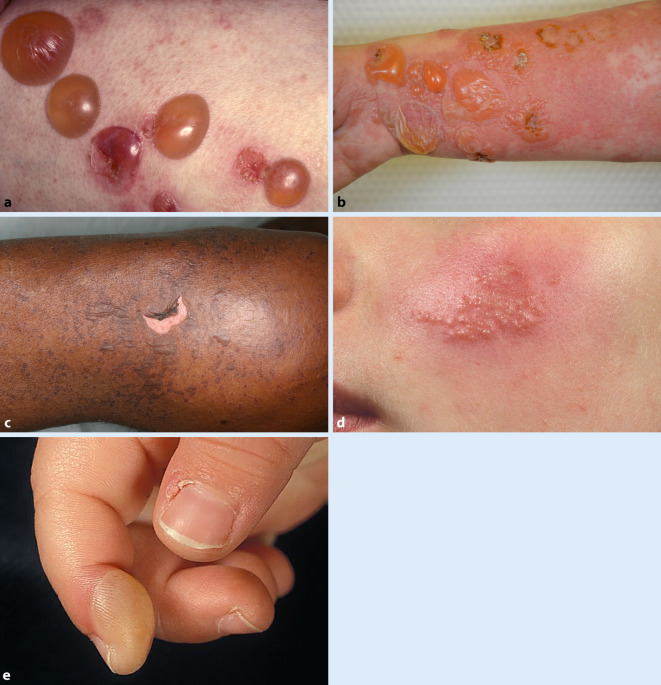

Im Laufe des Lebens kommt es vermutlich bei jeder Person zu dem Auftreten von Blasen [1]. Diese Blasen an der Haut können grundsätzlich durch sehr unterschiedliche Krankheitsbilder verursacht werden (Tab. 1). Somit handelt es sich hierbei um eine interdisziplinär und interprofessionell relevante Herausforderung (Abb. 1).

**Table 1:** 

*Hereditäre Erkrankungen*	Bullöse Ichthyosen
	Epidermolysis-bullosa-Gruppen
	Porphyrien
*Bullöse Autoimmundermatosen*	Bullöser Lichen planus
	Dermatitis herpetiformis Duhring
	Epidermolysis bullosa acquisita
	Graft-versus-Host-Krankheit
	Lineare IgA-Dermatose
	Pemphigoid-Gruppe
	Pemphigus-Gruppe
*Infektionserkrankungen*	Bulla repens
	Bullöse Tinea
	Bullöses Erysipel
	Hand-Fuß-Mund-Krankheit
	Herpes-Erkrankungen
	Impetigo contagiosa
	Staphylococcal-Scalded-Skin-Syndrom
*Physikalische Faktoren*	Druck
	Reibung
	Erfrierung
	Verbrennung
	Noxen
	UV-Licht
*Medikamente*	Fixes Arzneimittelexanthem
	Erythema exsudativum multiforme
	Steven-Johnson-Syndrom
	Toxisch epidermale Nekrolyse
*Sonstige Erkrankungen*	Artefakte
	Bullöse Insektenstichreaktionen (Culicosis bullosa)
	Bullosis diabeticorum
	Dyshidrosiforme Ekzeme/Cheiropodopompholyx

Bei der Fachgesellschaft Initiative Chronische Wunden (ICW) e. V. wurde angefragt, wie das therapeutische Vorgehen bei dem Auftreten von Blasen an der Haut sein sollte. Es fiel dann auf, dass bislang eindeutige Standards fehlen und die praktizierten Vorgehensweisen sehr unterschiedlich sind. Dies war der Anlass für den Vorstand der ICW, in Absprache mit dem wissenschaftlichen Beirat, sich mit dieser Fragestellung zu beschäftigen. Um Empfehlungen aussprechen zu können, wurde zuerst die wissenschaftliche Literatur kritisch durchgeschaut und im Hinblick auf praxisrelevante Empfehlungen geprüft. Anschließend wurden die dort gefundenen Empfehlungen in einem Konsensverfahren abgestimmt.

## Diagnostik und Einteilung

Blasen an der Haut können im Rahmen der unterschiedlichen Krankheitsbilder durch intrazelluläre Ödeme, Apoptose, Verlust desmosomaler Zellkontakte oder physikalische Zerstörung der Kontinuität verschiedener Hautbereiche entstehen [2]. Somit resultieren Blasen in unterschiedlicher anatomischer Tiefe der Haut, die dann entsprechend eingeteilt werden können (Tab. 2).

**Table 2:** 

Subkorneal
Intraepidermal
Subepidermal

Die Tiefe der Blase ist bereits ein erstes diagnostisches Zeichen, das durch klinische Inspektion und Palpation abgeschätzt werden kann. Subkorneale und intraepidermale Blasen platzen sehr schnell, sodass sie von den Betroffenen oft nicht als Blasen, sondern ausschließlich als Erosionen wahrgenommen werden. Die Abheilung erfolgt meist als Restitutio ad integrum und somit ohne Narbenbildung [3]. Hingegen ist das Blasendach bei subepidermalen Blasen deutlich dicker und somit fester. Wenn die Basalmembran der Epidermis zerstört wird, kommt es zu einer Defektheilung mit Narben, die zu Funktionseinschränkungen führen können. Bei einigen dieser Krankheitsbilder sollten Biopsien und, insbesondere für die Diagnostik bullöser Autoimmunerkrankung, auch eine direkte Immunfluoreszenzdiagnostik (DIF) sowie eine indirekte Immunfluoreszenzdiagnostik (IIF) durchgeführt werden [2]. Bei klinischem Verdacht auf Infektionskrankheiten oder sekundäre Weichgewebeinfektionen werden zudem serologische Parameter bestimmt, wie beispielsweise Blutbild und C‑reaktives Protein. Die weitere Diagnostik sollte dann gezielt entsprechend der jeweiligen Verdachtsdiagnosen durchgeführt werden.

In der klinischen Diagnostik von Blasen kann die Testung des Nikolski-Phänomens hilfreich sein. Der Typ 1 beschreibt die Erzeugung von Blasen durch seitlichen Druck auf die klinisch unverändert erscheinende Haut. Dieses klinische Zeichen ist beispielsweise bei Pemphigus vulgaris oder toxisch epidermaler Nekrolyse (TEN) positiv. Der Typ 2 beschreibt die Verschieblichkeit einer bereits bestehenden Blase innerhalb der Haut. Dieses Zeichen ist beispielsweise bei bullösem Pemphigoid positiv [2].

Die Diagnostik des Blaseninhalts kann insbesondere für den Nachweis von Erregern bei infektiologischer Genese sinnvoll sein.

Der sogenannte Tzanck-Test, synonym auch als Blasengrundzytologie bezeichnet, wird heute nur noch selten durchgeführt, obwohl es sich um ein einfaches und schnell durchzuführendes Verfahren handelt. Mit einem Skalpell wird Gewebematerial von dem Blasengrund abgeschabt, auf einen Objektträger aufgebracht, gefärbt und mikroskopisch untersucht. Der Test gilt als positiv, wenn verschmolzene akantholytische Keratinozyten als sogenannte vielkernige Riesenzellen nachgewiesen werden können. Somit ergibt sich dann ein Hinweis beispielsweise auf eine Herpeserkrankung oder einen Pemphigus vulgaris [4].

## Therapeutische Vorgehensweisen

Wenn es zu einer Blase an der Haut kommt, werden in klinischen Alltag verschiedene lokaltherapeutische Vorgehensweisen praktiziert. Entweder wird die Blase belassen, die Blase punktiert und das Blasendach belassen oder es wird das komplette Blasendach abtragen. Jede dieser Vorgehensweisen hat potenziell Vor- und Nachteile, die bei der Auswahl individuell berücksichtigt werden sollten (Tab. 3). Es sollte dabei beachtet werden, dass insbesondere bei Patient*innen mit einem diabetischen Fußsyndrom (DFS) aufgrund der Polyneuropathie Schmerzen oft fehlen [5].

**Table 3:** 

Vorgehen	Vorteile	Nachteile
*Blase belassen*	Geringe Infektionsgefahr	Ggf. Druckschmerz
	Einfacher bzw. kein Verband	Ggf. unkontrollierte Ruptur
	Geringe Kosten und Aufwand	Keine Entfernung des Blaseninhalts
		Keine Beurteilung des Blasengrundes
*Blase punktieren*	Einfacher Verband	Sterile Materialien
	Druckentlastung	Keine Beurteilung des Blasengrundes
	Entfernung des Blaseninhalts	Infektionsgefahr
*Blasendach abtragen*	Druckentlastung	Größte Infektionsgefahr
	Entfernung des Blaseninhalts	Sterile Materialien
	Beurteilung des Blasengrundes	Komplexerer Verband
		Regelmäßige Verbandwechsel
		Höhere Kosten und Aufwand

Nun stellt sich die Frage, ob eine dieser Vorgehensweisen für alle Arten von Blasen an der Haut empfohlen werden kann oder ob es vielleicht doch sinnvoll ist, das Vorgehen in Abhängigkeit von der Ätiologie, klinischer Faktoren, Komorbiditäten oder anderer Aspekte unterschiedlich zu empfehlen (Tab. 4). Für Patient*innen könnten zudem zahlreiche weitere Aspekte für die individuelle Auswahl des Vorgehend relevant sein (Tab. 5). Das letztendliche Vorgehen sollte daher im Vorfeld mit den Patient*innen besprochen und dann bei der partizipativen Entscheidungsfindung („shared decision making“ [SDM]) berücksichtigt werden [6].

**Table 4:** 

Diagnostik des Blaseninhalts sinnvoll, z. B. bei infektiöser Genese
Dicke des Blasendachs, z. B. bei intraepidermalen Blasen droht spontane Ruptur
Größe der Blase, z. B. führt zu funktioneller Einschränkung
Komorbiditäten, z. B. Immunsuppression mit erhöhter Sepsisgefahr
Lokalisation, z. B. droht spontane Ruptur
Schmerzhaftigkeit, z. B. bei sehr prall gefüllten Blasen
Ursache bzw. Pathomechanismus

**Table 5:** 

Aufwand bzw. Zeit bis zur vollständigen Abheilung
Infektionsrisiko
Kosmetische Resultate
Kosteneffizienz
Narbenqualität
Schmerzen

## Literatur

Es finden sich zu den lokaltherapeutischen Vorgehensweisen bei Blasen an der Haut sehr viele unterschiedliche Publikationen, die von der Autor*innengruppe hier exemplarisch vorgestellt und bewertet werden. Eine vollständige, kritische Bewertung der Gesamtliteratur zu dieser Thematik war nicht die Intention dieses Positionspapiers.

Im Tierversuch konnte gezeigt werden, dass bei intakter Blase der Wasserverlust durch Verdunstung von einer Verbrennungsoberfläche vergleichbar wie bei normaler Haut war. Wenn aber das Blasendach entfernt wurde, war die Wasserverlustrate anfänglich mehr als 100 × und später immer noch 20 × höher im Vergleich zu gesunder Haut. Diese hohe Wasserverlustrate war mit einer zunehmenden Tiefe der Wundzerstörung durch Austrocknung verbunden. Wurde die Blase intakt gelassen, war die Tiefe des Hautverlustes am geringsten und es kam zu einer verbesserten Heilung. Somit war es das Fazit der Autor*innen, Verbrennungsblasen, wann immer möglich, intakt zu lassen [7]. Hier ist anzumerken, dass die Daten, die aus diesem Tierversuch mit 100 Albino-Meerschweinchen und artifiziell induzierten Verbrennungsblasen schon 1976 zwar sehr interessante erste Erkenntnisse geliefert haben, aber sicher nicht 1:1 auf die Situation bei Menschen übertragen werden können.

In einer prospektiven randomisierten klinischen Studie wurden 40 Patient*innen mit Verbrennungsblasen > 6 mm in zwei Gruppen randomisiert. Hier erfolgte entweder die Punktion oder Abtragung des Blasendachs. Die durchschnittliche Anzahl der Tage bis zur vollständigen Wundheilung war vergleichbar, ebenso die Infektionsraten. Die Qualität der Narben und die Schmerzsymptomatik wurden in der Gruppe mit der Punktion tendenziell als besser bewertet [8]. Bei beiden Studien ist anzumerken, dass die Wunden nach Blasendachabtragung nicht mit hydroaktiven Wundauflagen versorgt wurden und somit ausgetrocknet sind. Heute gibt es für die Behandlung dieser Wundflächen moderne therapeutische Alternativen [9].

Ein frühes epidermales Débridement von Verbrennungen zweiten Grades führte in einer Studie im Tierversuch zu mehr Infektionen, langsameren Reepithelisierungsraten und zur Ausbildung von mehr Narbengewebe [10]. Auf der Basis einer Literaturrecherche wurden für Verbrennungen zweiten Grades einige Empfehlungen erarbeitet. Es wurde hier eine Differenzierung nach Größe und Wandstärke vorgenommen. Kleine Blasen (< 6 mm) sollten intakt belassen werden, weil es unwahrscheinlich ist, dass sie spontan aufplatzen, das darunterliegende Gewebe schädigen oder die Heilung behindern. Hingegen sollten große Blasen (> 6 mm) abgetragen werden, da sie mit größerer Wahrscheinlichkeit spontan aufreißen. Zudem wurde darauf hingewiesen, dass es durch den mechanischen Druck zu einer Schädigung des darunterliegenden Gewebes kommen kann [11]. Warum konkret bei 6 mm diese Differenzierung vorgenommen wird, wurde von den Autor*innen nicht weiter begründet.

Für die Behandlung von Wunden nach Verbrennungen gibt es erste wissenschaftliche Hinweise, dass der Einsatz der Vakuumtherapie („negative pressure wound therapy“, NPWT) verschiedene Aspekte der Wundheilung, insbesondere über eine Modulation der Inflammation, positiv beeinflussen kann [12].

Bei Blasen nach körperlicher Aktivität wurde auf der Basis klinischer Erfahrung die Punktion der Blasen ohne Entfernung des Blasendachs durch eine Expert*innengruppe empfohlen [13]. Begründet wurde dieses Vorgehen mit der besten Schmerzreduktion bei vergleichsweise geringem Infektionsrisiko.

Bei 53 Patient*innen mit Spannungsblasen an der Haut über Knochenfrakturen wurden randomisiert die 3 zuvor beschriebenen Vorgehensweisen getrennt voneinander untersucht. Wenn das Blasendach abgetragen wurde, erfolgte eine Behandlung mit Silbersulfadiazin-Creme. Bei den analysierten Ergebnissen konnte hinsichtlich der Endpunkte keine signifikanten Unterschiede zwischen den verschiedenen Modalitäten festgestellt werden [14]. Hier ist anzumerken, dass zumindest für Verbrennungen in einer Cochrane-Metaanalyse gezeigt werden konnte, dass Silbersulfadiazin die Wundheilung behindert und heute nicht mehr verwendet werden sollte [15].

### AWMF-Leitlinien

Die Leitlinien der Arbeitsgemeinschaft der Wissenschaftlichen Medizinischen Fachgesellschaften (AWMF) e. V. sind systematisch entwickelte Hilfen für Ärzt*innen und Patient*innen zur Entscheidungsfindung für eine angemessene Versorgung in spezifischen klinischen Situationen. Bei der Erstellung dieser Leitlinien sollten sowohl die bestmögliche wissenschaftliche Evidenz als auch die klinische Fachkompetenz berücksichtigt werden. Es erfolgte daher die kritische Durchsicht der potenziell relevanten AWMF-Leitlinien in Hinblick auf konkrete Empfehlungen für das lokaltherapeutische Vorgehen bei Blasen an der Haut.

S2k – Diagnostik und Therapie des Pemphigus vulgaris/foliaceus und des bullösen Pemphigoids: Es wird empfohlen, große bzw. mechanisch beeinträchtigende Blasen steril zu punktieren unter Erhalt des Blasendachs, das einen zusätzlichen Infektionsschutz darstellt [16].

S2k – Behandlung thermischer Verletzungen des Erwachsenen: Bei oberflächlicher zweitgradiger Verbrennung sollen Brandblasen […] entfernt werden […] da sie die Wundheilung beeinträchtigen.

Der definitiven Beurteilung der Verbrennungstiefe anhand des Wundgrundes sollen das Abtragen der ggf. entstandenen Hautblasen und die Reinigung der Wunde von Schmutz (Ruß etc.) vorausgehen.

Bei chemischen Verbrennungen sollen in der Klinik bestehende Blasen eröffnet werden, um eine sichere Dekontamination zu gewährleisten [17].

S2k – Thermische Verletzungen im Kindesalter (Verbrennung, Verbrühung), Behandlung: Es wird keine konkrete Empfehlung für das Vorgehen bei Blasen erwähnt [18].

## Diskussion

Es gibt nicht die eine richtige Vorgehensweise für die lokaltherapeutische Behandlung von Blasen an der Haut. Bei der Entscheidung über die jeweilige Vorgehensweise sollten daher verschiedene individuelle Faktoren und insbesondere die Genese berücksichtigt werden (Tab. 6). Sehr wichtig ist es dabei, immer auch zu berücksichtigen, dass hier verschiedene Ursachen für die Blasenbildung zugrunde liegen können, die dann sehr unterschiedliche Gesamttherapiekonzepte, beispielsweise mit Immunsuppression oder Virustatika, notwendig machen.

**Table 6:** 

Unkomplizierte Blasen ohne Druckschmerz: Blasen belassen
Druckschmerzhafte Blasen: Blasen punktieren und Dach belassen
Palmar und plantar lokalisierte Blasen: Blasen punktieren und Dach belassen
Rupturierte Blasen ohne klinische Infektionszeichen: Reste des Blasendachs belassen
Rupturierte Blasen mit klinischen Infektionszeichen: Reste des Blasendachs abtragen
Verbrennungsblasen ab Grad 2a: Blasendach abtragen
Blasen bei unklarer Verbrennungstiefe: Blasendach abtragen
Blasen bei chemischer Verbrennung: Blasendach abtragen

Wenn die Blase ohne weitere Intervention intakt belassen wird, ist meist keine spezifische Lokaltherapie erforderlich. Wenn die Blase punktiert und das Blasendach belassen wird, sollte eine trockene, sterile Abdeckung beispielsweise mit Baumwollkompressen oder einem Pflaster erfolgen. Wenn das Blasendach rupturiert ist oder abtragen wurde, sollte anschließend ein steriler Wundverband mit beispielsweise einer nichtadhäsiven (beschichteten) Gaze beziehungsweise einem Wunddistanzgitter angelegt werden. Für unproblematische Erosionen gibt es auch nichtadhäsive Pflaster [8].

**Figure 2:**
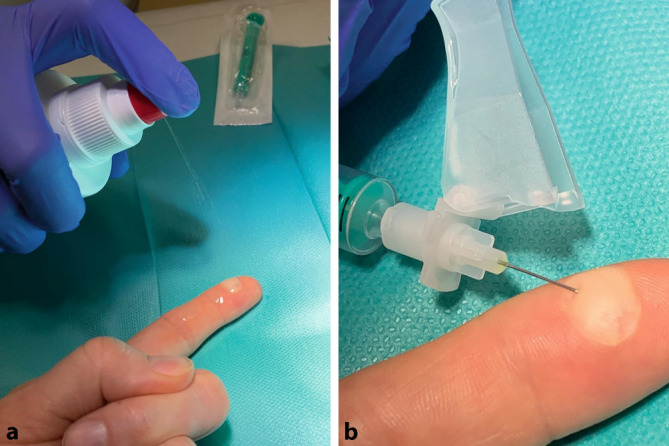

Für die Punktion von Blasen sollten ausschließlich sterile Instrumente wie beispielsweise Kanülen genutzt werden. Der Stich in die Blase sollte parallel und nicht senkrecht zur Hautoberfläche erfolgen, damit der Blasengrund nicht verletzt wird (Abb. 2).

Bei Verunreinigungen oder bakterieller Superinfektion sollten temporär zusätzlich antimikrobielle Wundprodukte, beispielsweise mit Octenidin oder Polihexanid, zum Einsatz kommen [19]. Für die klinische Diagnostik einer lokal infizierten Wunde und die somit resultierende Einschätzung der Notwendigkeit einer antimikrobiellen Therapie, kann der TILI-Score genutzt werden [20].

## Fazit für die Praxis

- Blasen an der Haut können sehr unterschiedliche Ursachen haben.
- Bei der Planung eines therapeutischen Konzepts sollte die Genese der Blasen diagnostiziert werden und ggf. eine kausale Behandlung erfolgen.
- Die Expert*innen der Initiative Chronische Wunden (ICW) e. V. haben nach Durchsicht der aktuellen Literatur und Konsensfindung konkrete Empfehlungen für die Lokaltherapie von Blasen ausgesprochen.
- Diese empfohlenen Vorgehensweisen orientieren sich an verschiedenen individuellen Faktoren und können somit interindividuell sehr unterschiedlich sein.
